# Tailoring the Formation of Functionalized Furans from Glucose in Water with Nature-Sourced Catalysts and In Situ NMR

**DOI:** 10.3390/molecules29061368

**Published:** 2024-03-19

**Authors:** Stefan S. Warthegau, Sebastian Meier

**Affiliations:** Department of Chemistry, Technical University of Denmark, Kemitorvet, Building 206, 2800 Kongens Lyngby, Denmark; sswa@kemi.dtu.dk

**Keywords:** carbohydrates, chain elongation, furan derivative, in situ NMR, Knoevenagel condensation, natural catalysts, sustainability

## Abstract

Chain elongation of unprotected carbohydrates in water under mild conditions remains a challenge both in chemical and biochemical synthesis. The Knoevenagel addition or condensation enables transformations to bioactive scaffolds for pharmaceutical and agrochemical compounds. Unfortunately, the catalysts in use for these transformations often reduce the green metrics of the transformations. Here, we use in situ NMR visualizations to explore the prospective use of natural catalysts for the synthesis of triple- and quadruple-functionalized furan- or dihydrofuran-derivatives from glucose and malononitrile. The dihydrofuran derivatives are formed as kinetic, major intermediates in the pathway to furan derivatives when using naturally abundant MgO or bio-sourced chitosan and *N*-Methyl-d-glucamine (meglumine) as the catalysts in water. Both catalyst loading, solvent composition and pH can be adapted to populate dihydrofurans with four substituents by slowing down their further reactions. Higher temperatures and higher pH values favor the formation of triple-functionalized furans over quadruple-substituted dihydrofurans, which may be bicyclic or monocyclic. Compared to more traditional catalysts, nature-sourced options offer more sustainable options that emulate natural processes. Visualization with in situ NMR contributes to streamlining the development of cheap and environmentally benign procedures for carbohydrate chain elongation.

## 1. Introduction

Carbohydrates, especially glucose monomers, are ubiquitous products of natural CO_2_ sequestration that hold great potential as sustainable building blocks for a future sustainable society [1,2,3,4,5,6,7,8]. While many dehydration [9,10,11,12,13] or isomerization [14,15,16,17,18,19,20] reactions seek to upgrade glucose to flexibly usable precursors either without modification of the carbon chain or by its cleavage, unlocking the full potential of carbohydrates as building blocks often requires extending their carbon chain [3,21,22,23]. Carbon chain extension hinges on C-C bond formation usually at the anomeric carbon, where the Knoevenagel reaction between an aldose and activated methylene groups offers a promising approach for selectively extending carbohydrate chains in water [24,25,26,27].

Heterogeneous catalysis of this transformation provides favorable metrics due to the easier purification of the catalyst from the products. The Knoevenagel reaction can use simple catalysts to forge new carbon–carbon bonds on bio-sourced molecules like glucose. This approach results in products with privileged scaffolds and documented use in the agrochemical, cosmetic and pharmaceutical industries [24]. Traditional Knoevenagel reactions often rely on catalysts such as aniline, piperidine and triethylamine [24,27,28], whose use is undesirable due to toxicity and environmental concerns. Nontoxic, robust and cheap catalysts sourced from nature are clearly preferred by chemical and pharmaceutical industries to comply with environmental requirements [29]. Such catalysts can ideally provide reusability and ease of separation. Hence, MgO has been suggested as a suitable heterogeneous catalyst for the conversion of glucose and malononitrile to dihydrogenated furan derivatives, albeit mechanistic details of the reaction have remained elusive [30].

Unveiling mechanistic details of transformations is crucial for optimizing and unlocking their full potential. Especially, NMR reaction tracking is well suited to visualize reaction progress in real-time with the goal of identifying kinetics and mechanisms, including the formation of transient reaction intermediates [31,32,33,34,35]. NMR observations using state-of-the-art equipment with high magnetic fields and cryogenically cooled detection electronics [36] can afford such detailed insight using high-resolution ^13^C NMR spectroscopy on reactions conducted with cheap reactants containing natural isotope distributions. Direct visualization of reaction progress thus affords efficient insight into the effects of conditions such as the temperature, pH, solvent or catalyst on the reaction outcomes.

**Figure 1:**
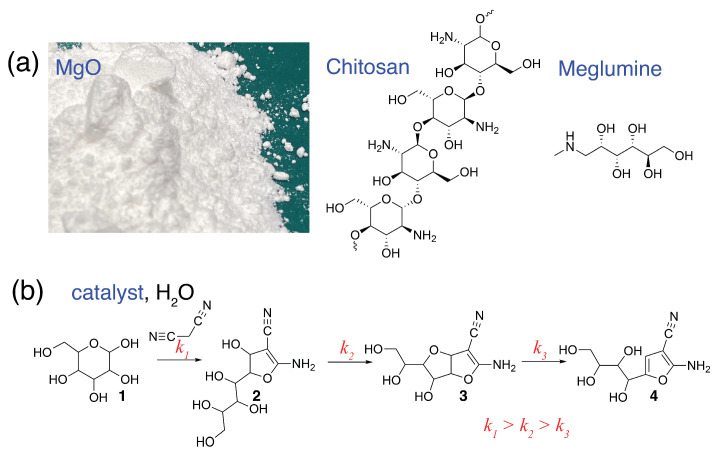
(**a**) Catalysts sourced from nature such as the heterogeneous catalysts MgO and chitosan (used at pH 11.0) and the glucose meglumine catalyze the reactions displayed in (**b**). Unprotected glucose (**1**) can be converted in water with these environmentally friendly catalysts to multiply substituted dihydrofuran (**3**) or furan (**4**) derivatives, subject to diligent choice of temperature, catalyst concentration and pH.

Here, we employed various nontoxic catalysts sourced from nature to achieve the C-C extension of unprotected glucose with malononitrile (Figure 1a) into substituted dihydrofuran or furan derivatives with high regio- and stereoselectivity. The catalysts included inorganic MgO and the reaction mechanism and active species in the reaction were scrutinized, yielding unanticipated results. Beyond MgO, organic chitosan was used at pH 11 as a heterogeneous catalyst for the successful formation of substituted dihydrofuran (Figure 1b, molecule **3**) or furan derivatives (**4**). As an environmentally friendly homogeneous catalyst derived from glucose, meglumine (*N*-Methyl-d-glucamine) was successfully employed for the C-C extension of unprotected glucose with malononitrile. The efficient formation of such densely functionalized furan derivatives in a single step in water is attractive compared to strategies requiring extensive workup between different steps [30]. Reaction mechanisms employing the different catalysts used herein prove to be similar and to hold surprises relative to the pathway suggested based on theoretical calculations, as they prove to proceed via the monocyclic dihydrofuran intermediate **2** (Figure 1b). Temperature and hydroxide ion concentrations were found to play a pivotal role in stabilizing additional unanticipated species such as bis-adducts of malononitrile to the aldehyde group. We expect that combining nontoxic catalysts, suitable high-resolution characterization techniques such as in situ ^13^C NMR and tuning of relevant reaction parameters affords efficient detections of benign reaction conditions for carbohydrate upgrading. The low cost and favorable environmental metrics of the investigated catalysts make their use in large-scale implementations plausible.

## 2. Results and Discussion

### 2.1. In Situ ^13^C NMR on MgO-Catalyzed Glucose Reaction with Malononitrile Clarifies Mechanism and Identifies the Kinetically Most Stable Intermediates

**Figure 2:**
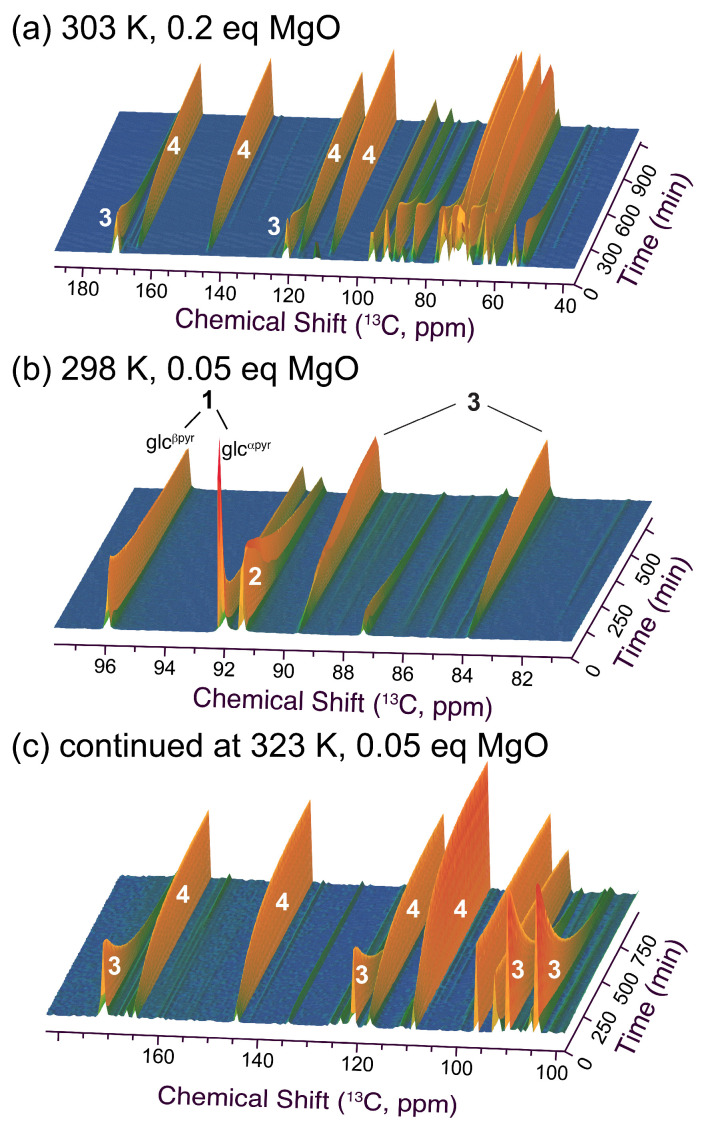
Time series of 1D-^13^C NMR spectra (time resolution of 4.8 min between individual spectra) for the conversion of glucose with malononitrile in the presence of MgO. Reaction conditions: 1 mmol glucose, 1 mmol malononitrile, MgO equivalents and temperatures were as indicated, 350 μL H_2_O/D_2_O (90%/10%).

Building on previous work describing the Knoevenagel reaction between aldoses and malononitrile using triethylamine catalysis [24,37], we started by tracking the reaction in the presence of MgO as the catalyst. MgO is a cheap and recyclable base catalyst that has been used both for glucose isomerization to fructose [15] and for the Knoevenagel reaction to the bicyclic dihydrofuran derivative **3** [30]. In the presence of 0.05 to 0.15 equivalents of MgO, low formation of the triple substituted furan derivative **4** to yields of 3–4% had been reported within 6 h at room temperature [30]. Hence, we were surprised to detect that a slight modification in the reaction conditions to 0.25 equivalents MgO and 303 K not only showed the rapid influx of reactants to **3** but also the sequential quantitative conversion to **4** in a reaction that proceeds slower than the formation of **3**, but it still reaches completion within 24 h (Figure 2a). Hence, the in situ observation indicated that the rapid formation of **3** and slower formation of **4** by elimination and aromatization are both viable under mild reaction conditions using commercial MgO as the catalyst.

The formation of **3** is sufficiently faster than its conversion to **4** to allow maximum accumulation of the intermediate on the order of 80%.

To gain additional mechanistic insight into the reaction, the conditions were modified to use 0.05 equivalents of the catalyst at 298 K (Figure 2b,c). Under these conditions, it was evident that mutarotation of the α-glucopyranose substrate to β-glucopyranose was paralleled by the Knoevenagel addition and rapid cyclization to **2** as previously observed for the triethylamine catalyzed conversion [37]. Hence, ring opening was a limiting step for the Knoevenagel addition under MgO catalysis. The intermediate **2** was converted to the bicyclic dihydrofuran derivative **3** within a few hours. Increasing the temperature to 323 K showed that full conversion to the triple substituted furan product **4** was viable even when using only 0.05 equivalents of MgO. Hence, we observed that MgO catalysis leads to rapid Knoevenagel addition, followed by 5-exo-dig cyclization to **2** and a slower dehydration to complete the Knoevenagel condensation and intramolecular oxa-Michael addition to form **3**. A slower elimination leads to the densely substituted heteroaromatic product **4**. This mechanism under MgO catalysis was somewhat surprising, considering that density functional theory computational calculations had previously indicated a different pathway [30]. Hence, the experimental observations based on in situ NMR underline the usefulness of experimental data to complement computational analyses of carbohydrate upgrading, where possible, due to the presence of various conceivable open-chain and cyclic forms of similar energy.

### 2.2. Filtration Test to Gain Insight on the Catalytically Active Species from MgO

**Figure 3:**
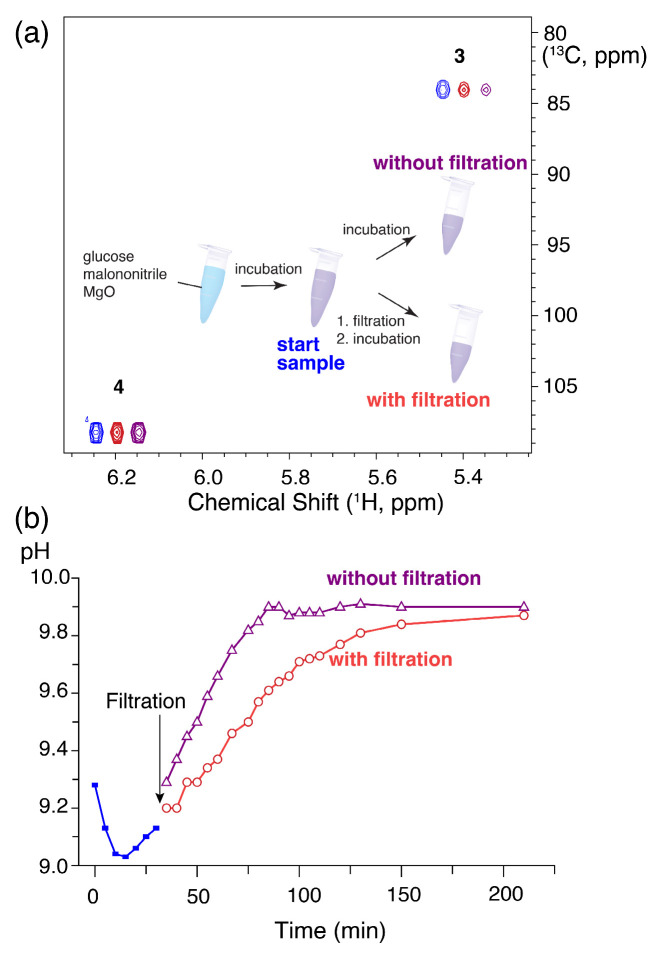
(**a**) Schematic depiction of a filtration test for the formation of **4** from glucose and malononitrile via intermediate **3** and ^1^H-^13^C HSQC spectrum showing signals for **3** and **4**. Conversion (loss of signal) of **3** from the start sample (blue) proceeded after removal of solid MgO from the reaction mixture (green), albeit to a lower degree than in the presence of solid MgO (purple). Reaction conditions: 1 mmol glucose, 1 mmol malononitrile, 0.2 eq MgO and 298 K, 350 μL H_2_O/D_2_O (90%/10%), 298 K. (**b**) pH change with and without filtration.

To gain additional insight into the catalytic process, a filtration test was subsequently employed. An ongoing reaction was split into two samples for comparison of further reaction progress in the presence or absence of solid MgO (Figure 3). Like the experiments displayed in Figure 2 showing the effect of different amounts of heterogeneous material, these experiments indicated that the solid MgO has a function in maintaining the conversion to product **4**. The conversion of **3** to **4** proceeded in the absence of solid material, but it was slower than for the sample containing solid MgO. These observations largely resemble observations for the role of MgO in the aldose-to-fructose isomerization of glucose to fructose [15], which indicated that MgO (with p*K*_sp_ = 9.8) provides OH^–^ ions through steady release and indicated a considerable contribution of homogenous catalysis to glucose isomerization in solution. The interpretation is somewhat jeopardized for the given reaction system, as malononitrile is converted to an amino group, hence inherently eliciting basicity and possibly also autocatalysis in the reaction of glucose and malononitrile. In the absence of filtration, however, pH increases more rapidly than upon filtration, thus corroborating the possibility that MgO acted as a source of slowly released hydroxides (Figure 3b).

**Figure 4:**
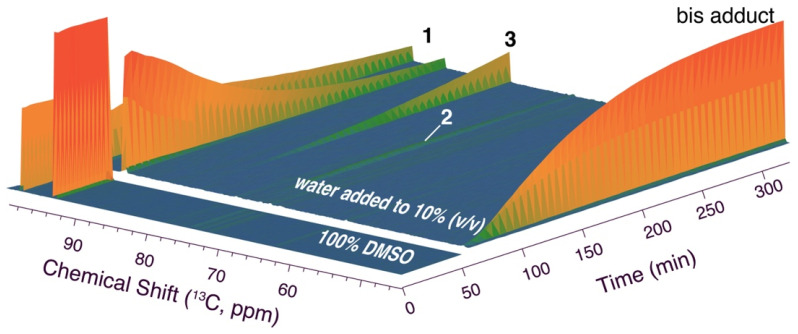
Time series of 1D-^13^C NMR spectra (time resolution of 7.2 min between individual spectra) for the conversion of glucose and malononitrile in the presence of MgO initially in the absence and then in the presence of added water (10% *v*/*v*). Molecules **1**, **2** and **3** correspond to glucose, monocylic dihydrofuran and bicyclic dihydrofuran, respectively, as displayed in Figure 1. Reaction conditions: 0.15 mmol [1-^13^C]glucose, 0.15 mmol malononitrile, 0.2 eq MgO and 313 K, 150 μL DMSO-*d_6_* or 167 μL DMSO-*d_6_*/D_2_O (90%/10%). Water favors the conversion of glucose and malononitrile, with low concentrations of water (10%) favoring the formation of a bis adduct of dihydrofuran rather than the formation of **2** and **3**.

**Scheme 1:**
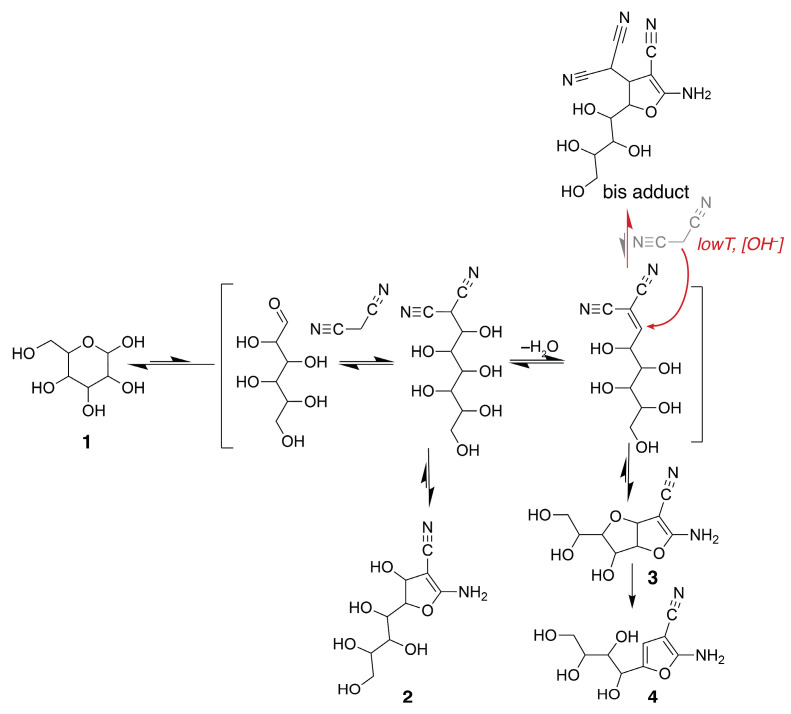
Schematic overview of the conversion of glucose (**1**) with malononitrile to dihydrofuran compounds **2** and **3** and furan derivative **4**, and the bis adduct using the naturally derived catalysts MgO, chitosan or meglumine. Product formation was affected by temperature, pH, water content and catalyst concentration.

This contribution of OH^–^ ions was further tested by employing DMSO as an aprotic medium in the conversion of glucose with malononitrile in the presence of MgO. MgO in DMSO did not yield any significant conversion of the reactants, while the addition of water to a final concentration of 10% (*v*/*v*) allowed the conversion of glucose and malononitrile to proceed to the dihydrofuran intermediates **2** and **3**. The low fraction of water in this sample of 10% (*v*/*v*) water/DMSO elicited a remarkable change in reaction progress; however, an adduct was formed where the C1 carbon was not carrying a hydroxyl group anymore, but rather, it yielded a carbon signal below 46 ppm as observed using isotope tracking with [1-^13^C]glucose (Figure 4). NMR spectroscopy validated that this species has twelve chemically non-equivalent carbon groups including three nitrile groups and one enamine, thus indicating that a bis adduct with the tentative structure shown in Scheme 1 was formed. The ^13^C chemical shifts of the species are at 170.5, 120, 112.7, 112.6, 86.0, 71.0, 70.1, 70.0, 62.9, 49.0, 45.5 and 28.1 ppm, with the signal at 45.5 ppm deriving from the glucose C1 position, consistent with a sp^3^ hybridized carbon not carrying an alcohol group. Bis adduct formation is well known to occur at the aldehyde group by Knoevenagel condensation and Michael addition, especially at ambient temperatures [27]. The observation that low [OH^−^] may favor the bis adduct formation is consistent with higher acidity of the activated methylene in malononitrile than of alcohol groups deriving from glucose. Higher deprotonation appears to result in a higher nucleophilicity of malononitrile than of alcohol groups in the Michael addition to the linear intermediate formed upon Knoevenagel condensation. The lack of water additionally should favor dehydration in the Knoevenagel condensation, thus populating the intermediate, to which the nucleophile attaches in a Michael addition.

### 2.3. Viability of a Glucose-Derived Catalyst: N-Methyl-d-Glucamine

**Figure 5:**
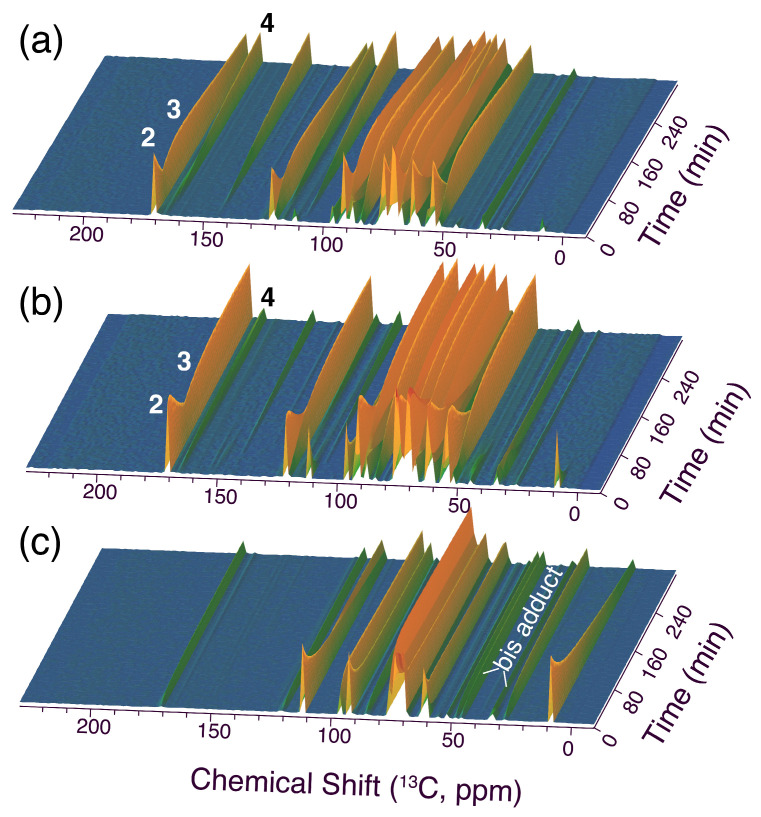
Time series of 1D-^13^C NMR spectra (time resolution of 2.4 min between individual spectra) for the conversion of glucose and malononitrile in the presence of meglumine. Molecules **2**, **3** and **4** correspond to monocylic dihydrofuran, bicyclic dihydrofuran, and the densely substituted furan, respectively (see Figure 1). Reaction conditions: 1 mmol glucose, 1 mmol malononitrile, 0.2 equivalents meglumine yielding (**a**), which was titrated from pH 11.0 to pH 9.4 and pH 7.6 for additional experiments ((**b**) and (**c**), respectively), 350 μL H_2_O/D_2_O (90%/10%), 308 K.

Considering the contribution of homogeneous catalysis to MgO catalysis, we resorted to identifying bio-sourced alternatives with low toxicity for catalyzing the conversion of glucose with malononitrile. *N*-Methyl-d-glucamine, also known as meglumine, is a glucose-derived secondary amine that is both bio-sourced and more environmentally friendly than other nitrogen bases commonly used for carbon chain extension. Conversion of glucose with malononitrile in aqueous solution with 0.2 equivalents meglumine at 308 K yielded the conversion shown in Figure 5a. The reaction mechanism and the intermediates and products formed strongly resemble the reaction progress under MgO catalysis. Hence, meglumine as a low-toxicity nitrogen base enabled the formation of **2**, the accumulation of **3** to approximately 80% selectivity and finally the conversion to the densely substituted furan **4**. To evaluate the role of hydroxide ions, the catalyst solution derived from dissolving meglumine in water (yielding pH 11.0) was titrated with 4 M HCl to pH 9.4 and pH 7.6 prior to addition of glucose and malononitrile. Using meglumine in the less basic catalyst solution (Figure 5b) yielded a more stable plateau for the formation of **3** as compared to higher pH (Figure 5a). This observation indicated that pH adjustments may be a promising strategy for regulating the reaction progress along the pathway shown in Scheme 1. An alternative way of achieving a stable formation of **3** was pursued by again using a DMSO/water mixture (90:10 *v*/*v*) and 0.2 equivalents of meglumine. The resultant reaction indeed predominantly yielded **3**, which proved stable in the reaction mixture at 308 K on a 24 h time scale (Figure S1). The conversion using meglumine at pH 7.6 validated that mechanistic changes can occur at low [OH^−^], as the bis adduct of malononitrile with the anomeric carbon of glucose accumulated at pH 7.6 under meglumine catalysis. The conversion of reactants at pH 7.6 proceeded more rapidly than anticipated due to the presence of an alternative pathway.

### 2.4. Catalysis by Chitosan

Encouraged by the successes in converting glucose and malononitrile with meglumine catalysis, we finally evaluated the prospect of using chitosan derived from the naturally abundant amino-polysaccharide chitin as a heterogeneous catalyst. Chitosan is very poorly soluble in aqueous solutions above pH 7 [38] and can serve as a heterogeneous catalyst at basic pH. Hence, commercial chitosan was suspended in water, and the pH was adjusted to 11.0 with 1 M NaOH. Subsequently, the chitosan was washed thrice in distilled water. The chitosan was then used for the conversion of equimolar glucose/malononitrile mixtures at 20 mg chitosan per mmol of the reactants, corresponding roughly to 0.12 equivalents on chitosan monomer—and hence amino-group basis.

**Figure 6:**
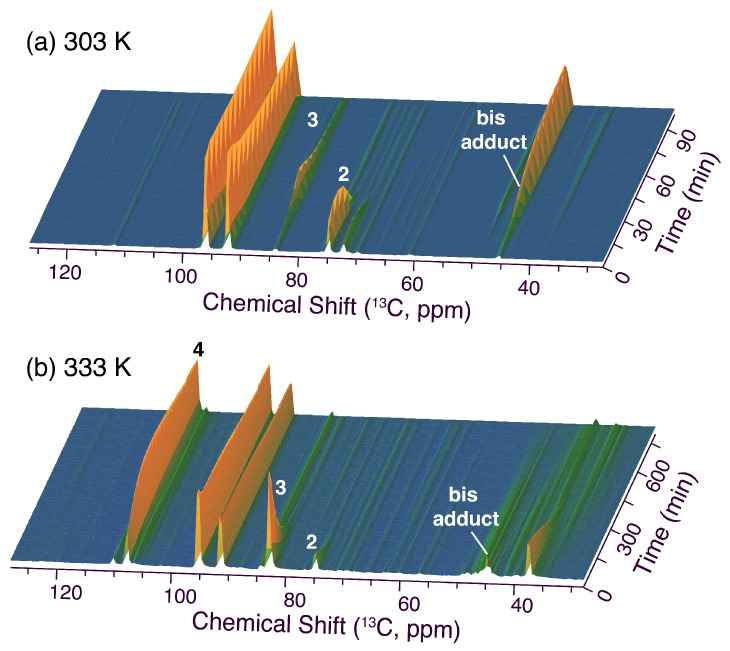
Time series of 1D-^13^C NMR spectra (time resolution of 7.5 min between individual spectra) tracking the conversion of [1-^13^C]glucose and 1 mmol malononitrile in a chitosan suspension (0.12 eq base equiv. on monomer basis) at (**a**) 303 K and (**b**) 333 K. Reaction conditions: 1 mmol [1-^13^C]glucose, 1 mmol malononitrile, 20 mg chitosan that was titrated with 1 M NaOH to pH 11.0 and washed thrice with distilled water to regenerate non-protonated amino groups, 350 μL H_2_O/D_2_O (90%/10%).

In situ ^13^C NMR reaction tracking of the reaction rapidly indicated that the reaction progress was slow at room temperature. A subsequent increase in temperature revealed that the reaction proceeded rapidly at 333 K as shown in Figure 6. The reaction proceeded via **2** and **3** to the densely substituted furan product **4**, with some bis adduct being formed during sample preparation and heating. This bis adduct could be converted at sufficiently high temperature, consistent with higher temperatures disfavoring the formation of the bis adduct relative to smaller adducts for entropic reasons. In the absence of a previous wash with alkaline solution to deprotonate the ammonium groups in chitosan, a more complex reaction progress was observed (Figure S2), again entailing a significant accumulation of higher adducts with malononitrile due to a low [OH^−^] in the reaction mixture near neutral pH. Nevertheless, the conversion to **4** was also viable with chitosan containing few free amino groups near neutral pH, albeit at lower selectivity than upon regeneration of free amines (Figure S1).

**Figure 7:**
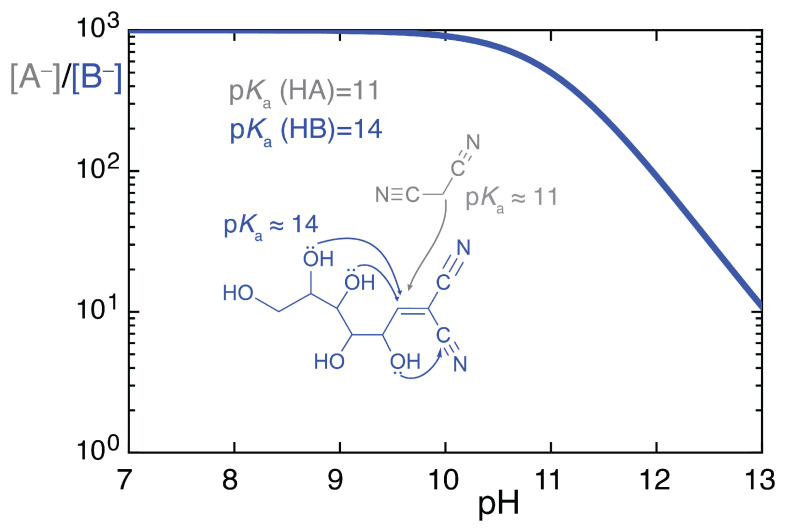
Ratio between a deprotonated electron-rich nucleophile (p*K*_a_ of 11) and a deprotonated electron-rich hydroxy groups (assuming a p*K*_a_ of 14) in dependence of pH, assuming equimolar malononitrile nucleophile and glucose in the starting material. Higher concentrations of deprotonated malononitrile at weakly basic pH can counteract entropic factors and the local availability of hydroxy groups that favor intramolecular oxa-Michael addition.

Overall, various nature-derived and environmentally benign catalysts can thus be used to form variously substituted furan rings from glucose and malononitrile. The hydroxy groups in glucose at secondary carbons are expected to have p*K*a values close to 14.0 [39,40], significantly weaker acidic than the CH acidic group in malononitrile with a p*K*a value close to 11.0 in water. Weakly basic conditions may hence render malononitrile the better nucleophile than intramolecular hydroxy groups in the Michael addition, converting the Knoevenagel addition product (shown in Figure 7). Deprotonated malononitrile is three orders of magnitude more likely deprotonated under these conditions than hydroxy groups with p*K*a values near 14.0. Hence, it may not be surprising that malononitrile can outcompete intramolecular hydroxy groups despite of the entropic cost and possibly steric reasons under suitable conditions. This effect is expected to fade for higher [OH^−^] values, which render hydroxy groups more likely deprotonated and electron rich (Figure 7), and at higher temperatures due to the entropic penalty associated with forming higher adducts. Hence, temperature, pH and solvent composition are promising factors for tweaking the formation of either triple-substituted furan or of dihydrofurans, which can be populated with triple substitution or with different quadruple substitutions.

## 3. Materials and Methods

### 3.1. Chemicals

Glucose (96%) and malononitrile (99%) were purchased from Sigma Aldrich (St. Louis, MA, USA). Isotope-enriched [1-^13^C]glucose was purchased from Sigma Aldrich, while deuterated water (D_2_O) and DMSO-*d6* were purchased from Aldrich. Chitosan with an average M_W_ of 20,000 Da was purchased from Glentham Life Sciences (Munich, Germany). *N*-Methyl-d-glucamine, MgO and Supelco buffer solutions were purchased from Sigma Aldrich.

### 3.2. General Reaction Procedure

The typical reaction procedure consisted of weighing glucose into a 1.5 mL Safe-lock Eppendorf (Hamburg, Germany) microcentrifuge tube to yield a final concentration of 2 M (in a 0.5 mL reaction) with 1.0 equivalent of malononitrile and 350 μL D_2_O for reactions in D_2_O, or in 300 μL deionized H_2_O and 50 μL D_2_O for reactions predominantly in protonated water. The reaction was started with the desired amount of catalyst. Commercial chitosan was suspended in water, and the pH was adjusted to 11.0 with 1 M NaOH. Subsequently, the chitosan was centrifuged, supernatant decarded, and the chitosan was washed thrice in distilled water to yield chitosan with regenerated free amino groups. *N*-Methyl-d-glucamine (0.2 mmol) was dissolved in 300 μL deionized H_2_O and 50 μL D_2_O, yielding a pH of approximately 11.0. Identical samples were produced and the solution of *N*-Methyl-d-glucamine was titrated to pH 9.4 and 7.6.

### 3.3. NMR Samples

For in situ NMR observations, the reaction mixture was transferred to a 5 mm NMR sample tube prior to tuning and matching, pulse calibration and manual shimming, followed by NMR spectroscopic observations. Reactions employing isotope-labeled [1-^13^C]glucose were conducted on a scale of 175 μL in 3 mm NMR tubes. Samples containing heterogeneous catalysts were spun in the NMR instrument at a frequency of 10 Hz to counteract the settling of the catalyst.

### 3.4. NMR Spectroscopy

All NMR spectra tracking of natural abundance reactants were acquired on an 800 MHz Bruker (Fällanden, Switzerland) Avance III instrument equipped with an 18.7 T magnet and a 5 mm TCI cryoprobe. NMR spectra tracking reactions with [1-^13^C]glucose were acquired on an 600 MHz Bruker Avance III instrument equipped with an 14.1 T magnet and a 5 mm Smartprobe. A time series of 1D ^13^C NMR spectra (zgig30) was implemented as a pseudo-2D experiment. An inter-scan relaxation delay d1 of 1.5 s was used, and 64–200 transients (depending on the sensitivity required) of the FID were accumulated. The FID was sampled during an acquisition time of 0.68 s. This approach results in a time resolution of 2.4–7.5 min as detailed in Figure legends to the displayed spectra. The stabilization of the sample by acid-quenching the *N*-Methyl-d-glucamine catalyzed reaction was verified using a 1D ^1^H NMR experiment employing excitation sculpting for water suppression (zgesgp pulse sequence) that was implemented as a pseudo-2D data set with two dummy scans and 16 transients per time point, an acquisition time of 1.3 s and an inter-scan relaxation delay of 1 s, resulting in a time resolution of 42 s. Multiplicity-edited ^1^H-^13^C HSQC (hsqcedetgpsisp.2) were employed to verify identifications of chemicals. All spectra were acquired, processed and analyzed using Bruker Topspin 3.5 pl6. Initial rates of malononitrile conversion in solutions of *N*-Methyl-d-glucamine of different pH but identical concentrations were obtained by fits in Bruker Topspin 3.5 pl6.

### 3.5. Filtration Test and pH Changes during the Reaction

In total, 12.0 mmol D-glucose (96%), 13.2 mmol malononitrile (99%) and 0.2 eq MgO, meglumine or triethylamine were added to a 15 mL screw cap tube and suspended in 6 mL demineralized water. When using MgO catalysis, datapoints of pH changes along the reaction trajectory were collected at room temperature with shaking of the reaction mixture prior to each data point. After 30 min, approximately 3 mL of the reaction mixture was vortexed and decanted to remove the catalyst. The remaining datapoints were collected using two different reaction mixtures separately, i.e., the filtered solution and the non-filtered reaction medium containing solid MgO. The pH-measurements were conducted using a Mettler Toledo (Columbus, OH, USA) FiveEasy F20-Basic pH meter which was calibrated using Supelco buffer solutions at pH 4 (B5020), pH 7 (B4770) and pH 10 (B4895). The pH measurement was repeated for reactions containing 12.0 mmol D-glucose (96%), 13.2 mmol malononitrile (99%) and 0.2 of either meglumine or triethylamine.

### 3.6. Data Plotting

Initial rates of malononitrile conversion in solutions of *N*-Methyl-d-glucamine of different pH but identical concentration and pH-dependent species distribution for *N*-Methyl-d-glucamine were plotted using pro Fit 7.0.19 (Quantum Soft, Uetikon am See, Switzerland). The time-dependent changes in pH during the reaction and during the filtration test were plotted in the same software.

## 4. Conclusions

Cheap, environmentally friendly catalysts for the chain elongation of glucose were readily identified using visualizations with high-resolution spectroscopy. Specifically, ^13^C NMR on state-of-the-art NMR instrumentation was suitable, as it capitalizes from the large chemical shift range and from the absence of solvent background and signal splitting while maintaining quantitative information.

The conversion of glucose and malononitrile to trisubstituted monocyclic furan **4** was catalyzed by naturally sourced, abundant, non-toxic and cheap catalysts such as MgO, chitosan or *N*-Methyl-d-glucamine. In all instances, the reaction proceeded via the trisubstituted monocyclic dihydrofuran **2** and quadruple substituted bicyclic dihydrofuran **3**. Identification of the chemical shifts for **2**, **3** and **4** will allow following the reaction in complex backgrounds and for various naturally sourced catalysts. Catalysis by MgO, chitosan or *N*-Methyl-d-glucamine led to rapid Knoevenagel addition followed by 5-exo-dig cyclization to **2**, a subsequently slower dehydration to complete the Knoevenagel condensation and intramolecular oxa-Michael addition to form **3**. A slower elimination led to the densely substituted heteroaromatic product **4**. The reaction of **3** to **4** is sufficiently slow relative to its formation to accumulate the bicyclic dihydrofuran **3** to approximately 80% of the maximum yield.

When using catalysis by MgO, the reaction between glucose and malononitrile was found to resemble glucose isomerization [15,41] insofar, as slow hydroxide release appeared to play a role in the catalysis that has contributions from homogeneous catalysis. The pH increased during the reaction converting a CH acidic nitrile to an enamine, thus also indicating the ability for the reaction to proceed autocatalytically (Figure S3). At low concentrations of dissolved hydroxide (in a 90:10 DMSO/water mixture) or at low concentrations of free chitosan or meglumine base (near neutral pH), the mechanism changed. A bis adduct of malononitrile at the anomeric center of glucose was populated at low hydroxide-ion concentrations and low temperatures, likely due to higher acidity and nucleophilicity of the malononitrile methylene group than of the hydroxy groups in competing reactions. Thus, solvent–catalyst interdependencies and their influence on reaction mechanisms became evident and can aid in developing sustainable processes. These results indicated that various bases catalyze the conversion of glucose and malononitrile along similar pathways, where control of solvent composition and pH, catalyst concentration and temperature afford the accumulation of either **3** or **4**. Abundant, nature-sourced catalysts can contribute to the environmental metrics for the carbon-chain extension of glucose, providing an optimistic outlook for the possibility to upscale glucose transformations to functionalized furans and dihydrofurans in aqueous solutions.

## Data Availability

The data presented in this study are available on request from the corresponding author.

## Supplementary Materials

The following supporting information can be downloaded at: https://www.mdpi.com/article/10.3390/molecules29061368/s1. Figure S1, Product mixture formed by meglumine catalysis in DMSO/water (90/10 *v*/*v*) at 308 K; Figure S2, Reaction progression in the presence of chitosan catalysis near neutral pH, yielding initial influx into bis adduct; Figure S3, pH changes during reaction progress at room temperature, validating pH changes due to the formation of an amino group.
