# Supplementary material for: Tailoring the Formation of Functionalized Furans from Glucose in Water with Nature-Sourced Catalysts and In Situ NMR
Source: Molecules. 2024 Mar 19;29(6):1368. doi: 10.3390/molecules29061368 (PMC10975964; doi:10.3390/molecules29061368)
Supplement: Supplementary file 1 [file molecules-29-01368-s001.zip › molecules-2926237-supplementary.pdf]

Supplementary Materials

Tailoring the Formation of Functionalized Furans from Glucose in Water with Nature-Sourced Catalysts and *In Situ* NMR

Stefan S. Warthegau[1] and Sebastian Meier[1,*]

[1] Department of Chemistry, Technical University of Denmark, Kemitorvet, Building 206, 2800 Kgs Lyngby, Denmark
* Correspondence: semei@kemi.dtu.dk

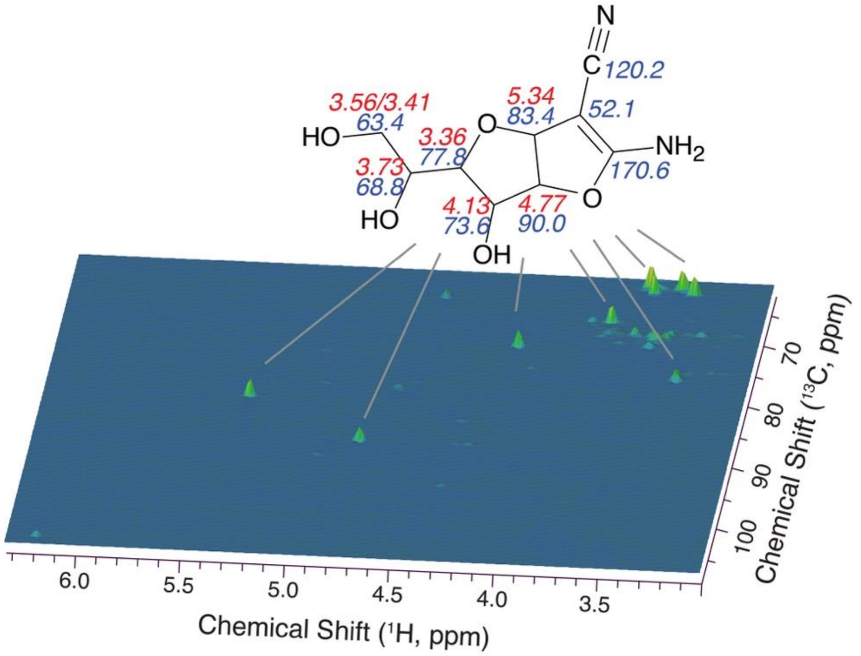

Figure S1. Conversion of 1 mmol glucose and 1 mmol malononitrile with 0.2 equivalents meglumine in 90% DMSO/10% D_2O yielded the shown bicyclic dihydrofuran **3** that was found stable at 308 K for 24 hours. Reaction conditions: 1 mmol glucose, 1 mmol malononitrile, 0.2 eq meglumine, 0.4 ml DMSO/D_2O (90%/10%), 333 K.

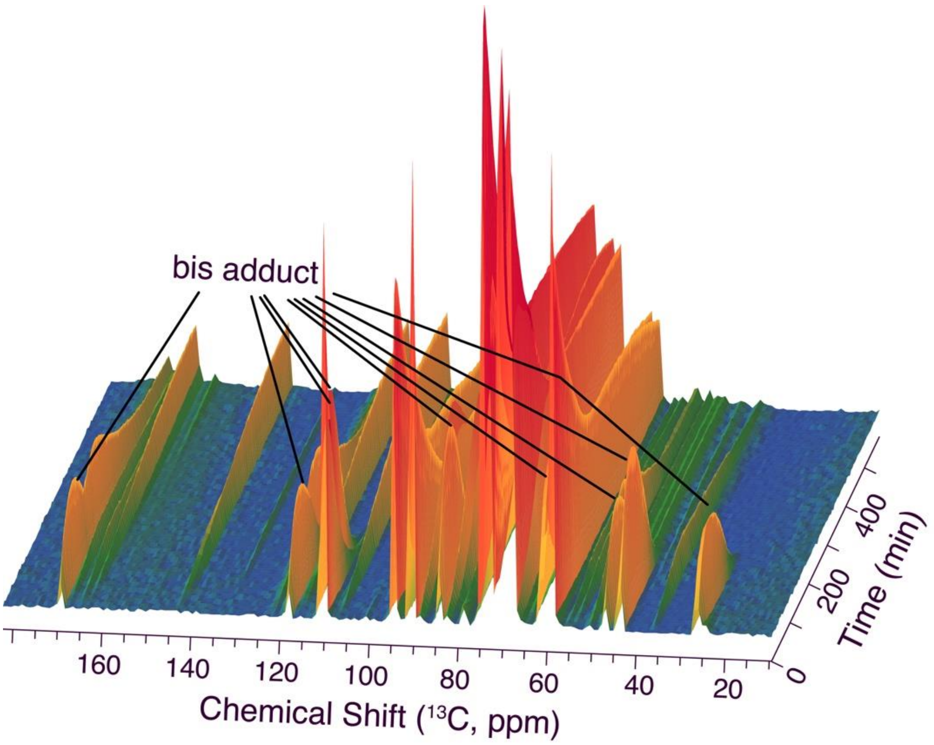

Figure S2. Series of 1D ^{13}C NMR spectra (time resolution of 4.8 min between individual spectra) for the conversion of 1 mmol glucose and 1 mmol malononitrile in a chitosan suspension (0.12 base equiv. on monomer basis). Reaction conditions: 1 mmol glucose, 1 mmol malononitrile, 20 mg chitosan without regeneration of free amino groups, 0.35 ml H_2O/D_2O (90%/10%), 333 K.

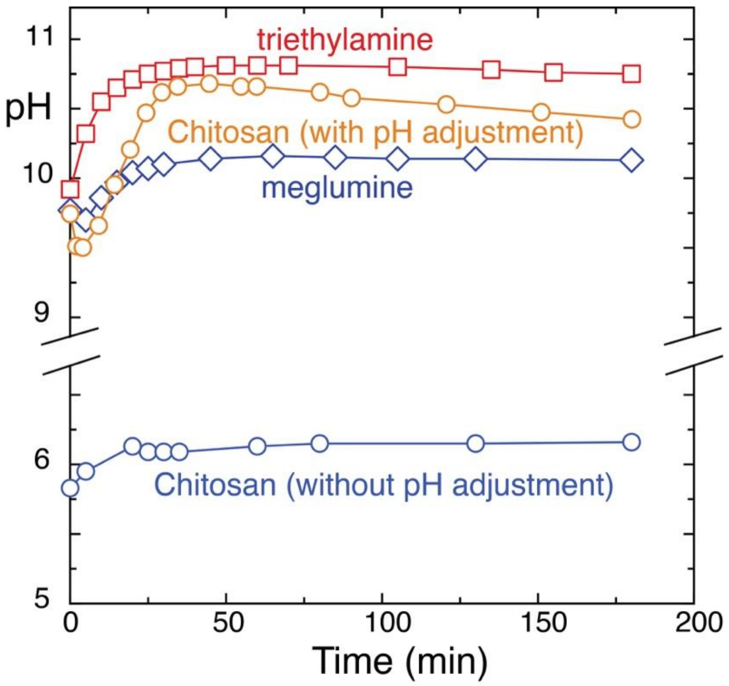

Figure S3. Changes in pH in the conversion of 1 mmol glucose and 1 mmol malononitrile in the presence of 0.2 equivalents conventional base (treithylamine), meglumine or chitosan (with or without initial pH adjustment to pH 11), showing slight increases in pH during reaction progress, likely due to the formation of an amino group.
